# Nondeterministic and co-Nondeterministic Implies Deterministic, for Data Languages

**DOI:** 10.1007/978-3-030-71995-1_19

**Published:** 2021-03-23

**Authors:** Bartek Klin, Sławomir Lasota, Szymon Toruńczyk

**Affiliations:** 8 University of Oxford, Oxford, UK; 9 Sorbonne Université - LIP6, Paris, France; University of Warsaw, Warsaw, Poland

**Keywords:** Data languages, register automata, determinizability, deterministic separability, sets with atoms, orbit-finite sets, nominal sets

## Abstract

We prove that if a data language and its complement are both recognized by nondeterministic register automata (without guessing), then they are also recognized by deterministic ones.

## 1 Introduction

Register automata are finite-state automata equipped with a finite number of registers that can store values from an infinite data domain. When processing an input string, an automaton compares the current input data value to its registers and, based on this comparison and on the current control state, it chooses its next control state and possibly stores the input value in one of its registers. In the original model, introduced over 25 year ago by Francez and Kaminski [15], data values can only be compared for equality and not for any other property. Subsequent extensions of the model allow for comparing data values with respect to some fixed relations such as a total order, or introduce alternation, variations on the allowed form of nondeterminism, etc.

It appears that register automata lack most of the good properties known from the classical theory of finite automata. For example, while languages of nondeterministic register automata are closed under unions and intersections, they are not closed under complement, and they do not determinize. Moreover, the expressivity of register automata is very sensitive to natural variants and extensions. Any of the following relaxations of the model leads to a strict increase of expressive power (see [15,23,1] for details):

– increasing the number of registers (when this number is bounded),
– extension from one-way to two-way automata,
– extension from deterministic to unambiguous, nondeterministic or alternating ones,

[†] Supported by the European Research Council (ERC) under the EU Horizon 2020 programme (ERC consolidator grant LIPA, agreement no. 683080).
[‡] Supported by the NCN grant 2019/35/B/ST6/02322.
[§] Supported by the NCN grant 2017/26/D/ST6/00201.

– adding the capability to nondeterministically guess data values.

In fact, almost every combination of these extensions leads to a different class of recognized languages. Furthermore, no satisfactory characterizations of languages of register automata in terms of regular expressions [17,20] or logic [23,12] are known. There are a few positive results: a simulation of two-way nondeterministic automata by one-way alternating automata with guessing [1], a Myhill-Nerode characterization of languages of deterministic automata [16,4,5], and the well-behaved class of languages definable by orbit-finite monoids [2], which admits equivalent characterisations in terms of logic [11] and a syntactic subclass of deterministic automata [7]. Nevertheless, register automata satisfy almost no semantic equivalences that hold for classical finite automata.

*Contribution.* Our primary contribution is a collapse result: if a language and its complement are both recognized by nondeterministic register automata (NRA), then they are both recognized by deterministic ones (DRA). In symbols, we prove the following equality of language classes:

$$\mathsf{NRA} \cap \mathsf{co\text{-}NRA} \quad = \quad \mathsf{DRA}.$$

This result is shown under the assumption that the data values can be compared only for equality, and it turns out to be quite fragile. For instance, it fails if the automata can compare data values using a total order relation. It also fails if NRA are additionally equipped with the capability of guessing fresh data values, even when data values can only be compared for equality.

Our secondary contribution is a collapse result for NRA with 1 register only (1-NRA), but over an arbitrary data domain that *admits well quasi-order* (WQO), meaning roughly that finite induced substructures of the data domain, ordered by embeddings, form a WQO. This includes both equality and ordered data domains. In short, we prove the following inclusion of language classes:

$$\mathsf{1\text{-}NRA} \cap \mathsf{co\text{-}1\text{-}NRA} \quad \subseteq \quad \mathsf{DRA}.$$

The inclusion is strict, as some DRA languages are not recognizable by 1-NRA.

Our proofs are mostly self-contained, but use basic notions and results about sets with atoms [1], also known as nominal sets [24]. In particular, automorphisms of the data domain play a central role in our arguments, and we extensively use notions such as finite support and orbit-finiteness of sets. In both results, we prove that for every data language $L \in \mathsf{NRA} \cap \mathsf{co\text{-}NRA}$ the set of derivative languages $w^{-1}L$ is orbit-finite, i.e., finite up to automorphism of data values. The collapse then follows from an orbit-finite version of the Myhill-Nerode theorem.

In our primary contribution, orbit-finiteness of the set of derivative languages is a consequence of a key technical result (Lem. 1), an abstract observation about orbit-finite families of sets, which we believe may be of independent interest. As another example application of this lemma, we give a new proof of decidability of universality for unambiguous register automata (URA).

*Relation to other work.* Our primary result partially confirms a conjecture of Thomas Colcombet [10], according to which every two disjoint languages of NRA with guessing are separable by a language recognized by an URA. Working in the special case when the NRA are complementing and have no guessing, we show more: both languages are then recognized not only by an URA but by a DRA.

NRA do not have good algorithmic properties: while the emptiness problem is PSpace-complete [14], the universality problem (does a given automaton accept all data words?) is undecidable [15] (it is decidable only for 1-NRA [14]). Universality becomes decidable for URA, as shown recently in [22] (2-ExpSpace upper bound, improved to 2-ExpTime upper bound in [8]), and language containment and equality for URA reduce polynomially to universality (see [8, Lemma 8]). As mentioned above, our results allow us to re-prove this decidability result.

Register automata have been intensively investigated, with respect both to their foundational properties [15,25,17,23] and to their applications to XML databases and logics [14] (see [26] for a survey). There are several other ways to extend finite-state machines with a capability to recognize languages over infinite alphabets. These include, apart from register automata: their abstract version – nominal automata or automata over atoms [4,5,1]; symbolic automata [13]; pebble automata [21]; and data automata [3,6].

*Acknowledgments.* We thank Lorenzo Clemente for posing the collapse question studied in this paper, and Joanna Ochremiak and Radek Piórkowski for valuable discussions.

## 2 Data languages and register automata

The model of register automata, as considered in this paper, is parametrized by an underlying relational structure Atoms over a finite vocabulary $\Sigma$. This structure constitutes a *data domain*; its elements are called *atoms*. A register automaton processes sequences of atoms, possibly coupled with labels from a fixed finite set. It may store atoms read from the input in its registers, and compare them with previously stored atoms using relations in $\Sigma$ (equality included).

Here are some example data domains:

- *Equality atoms*: natural numbers with equality $(\mathbb{N}, =)$. Since equality is the only available relation, any other countably infinite set could be used instead.
- *Dense order atoms*: rational numbers with the standard order $(\mathbb{Q}, \leqslant)$. Again, any countably infinite dense order without endpoints could be used instead.
- *Nested equality atoms* (universal equivalence relation): $(\mathbb{N}^2, =_1, =)$ where $=_1$ is the equality on the first coordinate: $(n_1, n_2) =_1 (m_1, m_2)$ if $n_1 = m_1$.

In the following we consider input alphabets of the form $S \times$ Atoms, where $S$ is a finite set of labels. A *data word* is a finite sequence $w \in (S \times \text{Atoms})^*$, and a *data language* is a set of data words.

A *nondeterministic register automaton* (NRA) $\mathcal{A}$ consists of:

- an input alphabet of the form $S \times$ Atoms, for some finite set $S$,

- a positive integer $r \in \mathbb{N}$ (the number of registers),
- a finite set of control states (locations) $Q$,
- subsets $I, F \subseteq Q$ of initial resp. accepting states,
- a finite set $\Delta$ of transition rules of the form

$$(p, s, \varphi, \textsc{st}, q) \in \Delta, \tag{1}$$

where $p, q \in Q$, $s \in S$, $\varphi(x_1, \dots, x_r, x)$ is a quantifier-free $\Sigma$-formula with free variables in $\{x_1, \dots, x_r, x\}$, and $\textsc{st} \in \{1, \dots, r, \textsc{none}\}$.

Intuitively, $\varphi$ defines a condition which needs to be satisfied by the register contents $(x_1, \dots, x_r)$ and by the current atom $(x)$ for a transition to happen, and $\textsc{st}$ specifies the register in which the input atom is stored after the transition, $\textsc{st} = \textsc{none}$ meaning that it is not to be stored in any register.

An NRA $\mathcal{A}$ is *deterministic* (DRA) if it has exactly one initial state and if for every two transition rules

$$(p, s, \varphi_1, \textsc{st}_1, q_1),\ (p, s, \varphi_2, \textsc{st}_2, q_2) \in \Delta,$$

such that $\varphi_1 \wedge \varphi_2$ is satisfiable in $\textsc{Atoms}$, we have $\textsc{st}_1 = \textsc{st}_2$ and $q_1 = q_2$. We write $r$-NRA, resp. $r$-DRA, when the number of registers $r$ is fixed.

A configuration $q(\mathbf{a}) \in Q \times (\textsc{Atoms} \cup \{\bot\})^r$ of $\mathcal{A}$ consists of a control state $q \in Q$ and a content of registers $\mathbf{a} \in (\textsc{Atoms} \cup \{\bot\})^r$, where $\bot$ means that the content of a register is undefined (i.e., the register is empty). A rule (1) induces a transition $p(\mathbf{a}) \xrightarrow{(s,a)} q(\mathbf{b})$ from a configuration $p(\mathbf{a})$ to a configuration $q(\mathbf{b})$ if:

- $\textsc{Atoms}, (\mathbf{a}, a) \models \varphi$ (by definition, this fails if $\varphi$ refers to any variable that has the undefined value $\bot$ in $\mathbf{a}$), and
- $\mathbf{b}$ is obtained from $\mathbf{a}$ by placing $a$ on coordinate $\textsc{st}$ if $\textsc{st} \neq \textsc{none}$, and $\mathbf{b} = \mathbf{a}$ otherwise.

A *run* of $\mathcal{A}$ on a data word $w = (s_1, a_1) \cdots (s_n, a_n)$ is a sequence

$$q_0(\mathbf{a}_0) \xrightarrow{(s_1,a_1)} q_1(\mathbf{a}_1) \xrightarrow{(s_2,a_2)} \dots \xrightarrow{(s_n,a_n)} q_n(\mathbf{a}_n),$$

where $q_0$ is an initial state and $\mathbf{a}_0$ is a tuple where the content of all registers is undefined. We then say that the configuration $q_n(\mathbf{a}_n)$ is *reachable along* $w$. The finite set of all configurations reachable along $w$ is finite, and it is denoted $\mathcal{A}(w)$.

A run is *accepting* if it ends in a configuration with an accepting state. A data word $w$ is *accepted* by $\mathcal{A}$ if there is an accepting run of $\mathcal{A}$ on $w$. A NRA is *unambiguous* (URA) if every word has at most one accepting run.

The *language* of $\mathcal{A}$, denoted $L(\mathcal{A})$, is the set of all data words accepted by $\mathcal{A}$.

## 3 Examples

In all our examples, the finite component $S$ of data alphabets will be a singleton set. We will therefore omit $S$ when describing automata, so (1) will simplify to

$$(p, \varphi, \textsc{st}, q) \in \Delta.$$

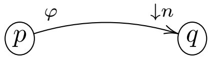

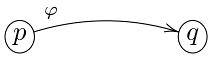

Graphically, a transition rule like this will be presented as if ST $= n$, and if ST $=$ NONE.

Furthermore, $\longrightarrow (p)$ means that $p$ is initial and $((q))$ means that $q$ is accepting.

*Example 1.* For the equality atoms, consider the language $L \subseteq \text{Atoms}^*$ of those words where the first letter appears at some later position:

$$L = \{a_1 \ldots a_n \mid n > 1, a_1 = a_i \text{ for some } i > 1\}.$$

This language is recognized by a DRA with one register and three control states:

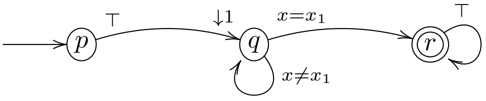

This automaton stores the first letter in its only register and then remains in the (non-accepting) state $q$ until the letter is encountered again; then it moves to the accepting state $r$ and stays there.

*Example 2.* Still for the equality atoms, consider the *reverse* of the language from Example 1, i.e., the language of those words where the last letter appears at some earlier position. This language is not recognized by any DRA, but it is recognized by a NRA with one register and three control states:

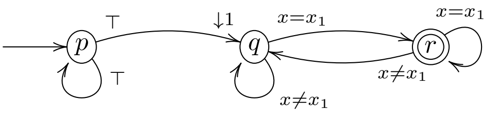

This automaton nondeterministically decides to store a letter in its register and then checks that the last letter is equal to the stored one.

*Example 3.* Still for the equality atoms, consider the *complement* of the language from Example 2, i.e., the language $L$ of those words where the last letter does *not* appear at any earlier position. (In particular, we consider the empty word and all length-one words to be in this language.)

The language $L$ is not recognized by any NRA. However, it becomes recognizable if automata are additionally equipped with the ability of *guessing*, that is, of updating the contents of their registers with arbitrary atoms, possibly different from the one that comes with the current input letter. Unlike NRA without guessing, those with guessing are closed under reversal [18, Def. 3 and Corollary 31], and the reversal of the language $L$ is even recognized by a DRA.

*Example 4.* Automata from Ex. 1-3 work just as well over the dense order domain: the formulas in their transition rules simply do not use the order relation. However, over densely ordered atoms something more happens: the language from Ex. 3 is recognizable by a NRA without guessing.

The automaton has two registers. The idea is that, at any moment in an accepting run where these registers store atoms $a_1 < a_2$:

(a) in the part of the word read so far, no letter is in the open interval $(a_1, a_2)$,
(b) the last letter of the word will belong to that open interval.

Condition (a) can be ensured easily: upon reading a letter $a$ that belongs to the open interval $(a_1, a_2)$, the automaton will (enter an accepting state for the moment and) put $a$ in one of the two registers. The register is chosen nondeterministically so that condition (b) remains true. If the currently input letter is not in the interval $(a_1, a_2)$, the automaton enters a rejecting state for the moment, with the registers kept unchanged.

Special treatment is needed to deal with situations where the last letter of the word will be larger than (or smaller than) all the letters encountered so far. These are taken care of by introducing special control states where one of the two registers remains undefined.

*Example 5.* Fix $k \geqslant 2$. Over equality atoms, consider the language $L_k$ of all words $w$ of length at least $k$ whose $k$th last letter is equal to the last letter. Then $L_k$ is recognised by a $\mathsf{NRA}$ with one register and $k+1$ states, depicted below:

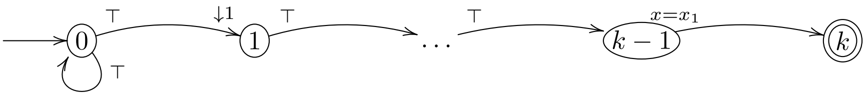

The complement of $L_k$ is also recognised by an $\mathsf{NRA}$, similar to the one above, but with $x \neq x_1$ in place of $x = x_1$ in the last transition, and with an additional component for accepting words of length smaller than $k$. The language $L_k$ is also recognised by a $\mathsf{DRA}$ with $k$ registers, where register number $i$ stores the letter which appeared on the latest seen position with index congruent to $i$, mod $k$. It has $k$ states, for counting the index of the current position, mod $k$.

## 4 Main results

Our primary contribution is:

**Theorem 1.** *Over equality atoms, if a data language and its complement are both recognizable by nondeterministic register automata, then they are both recognizable by deterministic register automata.*

Note that this result fails if automata with guessing are considered (see Ex. 3). Indeed, the language from Ex. 2 is recognized by a 1-$\mathsf{NRA}$, and its complement in Ex. 3 is recognized by a 1-$\mathsf{NRA}$ with guessing, but they are not deterministically recognizable.

Moreover, the result fails (even without guessing) for densely ordered atoms. The counterexample is the same: the language from Ex. 2 is recognized by a 1-$\mathsf{NRA}$, and its complement is recognized by a 2-$\mathsf{NRA}$ over densely ordered atoms as explained in Ex. 4, but they are not deterministically recognizable. Here the use of two registers in $\mathsf{NRA}$ is necessary, due to our secondary contribution: for a wide range of data domains, if a data language and its complement are both recognized by 1-$\mathsf{NRA}$, then they are recognized by $\mathsf{DRA}$.

We prove this for any data domain Atoms which admits wqo in the following sense. A *well quasi-order* (wqo) is a quasi-order $(Z, \leqslant)$ such that for every infinite sequence $z_1, z_2, \ldots \in Z$ there are $1 \leqslant i < j$ with $z_i \leqslant z_j$. For a finite set $X$, an $X$-labeled substructure of Atoms is a set $\mathcal{B} \subseteq$ Atoms together with a labelling $\ell_{\mathcal{B}} \colon \mathcal{B} \to X$. For two $X$-labeled substructures $\mathcal{B}$ and $\mathcal{C}$ of Atoms, we say that $\mathcal{B}$ embeds into $\mathcal{C}$ (written $\mathcal{B} \preceq \mathcal{C}$) if some automorphism $\pi$ of Atoms, restricted to $\mathcal{B}$, yields a label-preserving injection from $\mathcal{B}$ to $\mathcal{C}$, so that $\ell_{\mathcal{B}} = \ell_{\mathcal{C}} \circ \pi \restriction_{\mathcal{B}}$. Let $\text{age}_X(\text{Atoms})$ be the set of all finite labeled substructures of Atoms, partially ordered by $\preceq$. We say that Atoms *admits* wqo if for every finite set $X$, the quasi-order $(\text{age}_X(\text{Atoms}), \preceq)$ is a wqo. All data domains listed in Section 2 admit wqo [19]. They are also *oligomorphic* (see Sec. 5 below).

**Theorem 2.** *Over any oligomorphic atoms that admit* wqo*, if a data language and its complement are both recognizable by nondeterministic register automataa with one register, then they are recognizable by deterministic register automata.*

The rest of the paper consists of the proofs of Thms. 1 and 2, in Sec. 6 and 8, respectively, preceded by Sec. 5 that recalls basic definitions of the setting of sets with atoms which are used in the proofs. Our main technical lemma is proved in Sec. 6. Besides proving Thm. 1, in Sec. 7 we explain how it implies decidability of universality for unambiguous register automata.

## 5 Orbit-finite automata

Our proofs rely on some basic notions and results of the theory of sets with atoms [1], also known as nominal sets [24]. In this section we recall what is necessary to follow our arguments; this is part of a uniform abstract approach to register automata developed in [4,5,1].

Let Aut(Atoms) denote the group of all automorphisms of a relational structure Atoms. (For the equality atoms $(\mathbb{N}, =)$ this means the group of all bijections; for the densely ordered atoms $(\mathbb{Q}, \leqslant)$, the group of monotone bijections.) We consider sets equipped with an action of this group, typically, $\text{Atoms}^n$ for some $n \geqslant 0$ or $\text{Atoms}^*$ with the componentwise action.

*Group actions.* A (left) action of a group $G$ on a set $X$ is a mapping $\_ \cdot \_ :$ $G \times X \to X$ such that $1 \cdot x = x$ and $\sigma\pi \cdot x = \sigma \cdot (\pi \cdot x)$ for all $\sigma, \pi \in G$ and $x \in X$. We then say that $G$ *acts* on $X$, or that $X$ is a *G-set.* For $x \in X$, we call the set $\{\pi \cdot x \mid \pi \in G\}$ the orbit *of* $x$; or an orbit *in* $X$. The orbits in $X$ partition $X$ into disjoint sets. We call $X$ *orbit-finite* if it has finitely many orbits.

Group actions canonically extend along familiar set-theoretic constructions: if $X$ and $Y$ are $G$-sets then the cartesian product $X \times Y$, the disjoint union $X \uplus Y$, the set of sequences $X^*$, the powerset $\mathcal{P}(X)$ etc. are all $G$-sets, in the expected way. For example, $G$ acts componentwise on $X \times Y$ via $\pi \cdot (x, y) = (\pi \cdot x, \pi \cdot y)$.

*Oligomorphicity.* A structure Atoms is *oligomorphic* if for every $n \in \mathbb{N}$, the componentwise action of Aut(Atoms) on $\text{Atoms}^n$ induces finitely many orbits. All structures considered in this paper are oligomorphic; an example of a non-oligomorphic structure is the total order of integers.

*Supports.* Let $\mathrm{Aut}(\textsc{Atoms})$ act on a set $X$ and let $x \in X$. A *support* of $x$ is any set $S \subseteq \textsc{Atoms}$ such that the following implication holds for all $\pi \in \mathrm{Aut}(\textsc{Atoms})$:

if $\pi(s) = s$ for all $s \in S$ then $\pi \cdot x = x$.

An element $x \in X$ is *finitely supported* if it has some finite support.

For many structures $\textsc{Atoms}$, finite supports of a fixed element are always closed under intersections. Then every finitely supported $x$ has *the least support*, denoted $\sup(x)$. This happens in particular for the equality atoms (as proved in [24, Prop. 2.3] or in [5, Cor. 9.4]) and for the dense order atoms (as proved in [5, Prop. 9.5]). It is easy to prove that taking least supports commutes with group actions: $\pi \cdot \sup(x) = \sup(\pi \cdot x)$ for every $x \in X$ and $\pi \in \mathrm{Aut}(\textsc{Atoms})$.

*Equivariance.* An element (or a subset, relation, function...) of an $\mathrm{Aut}(\textsc{Atoms})$-set is called *equivariant* if it is supported by the empty set; equivalently, it is fixed by every automorphism of $\textsc{Atoms}$. For example:

- a subset $Z$ of an $\mathrm{Aut}(\textsc{Atoms})$-set $X$ is equivariant if and only if it is a union of orbits in $X$ (indeed, it is then equivariant as an element of $\mathcal{P}(X)$);
- a relation $R \subseteq X \times Y$ is equivariant if and only if $xRy \leftrightarrow (\pi \cdot x)R(\pi \cdot y)$ for all $x \in X$, $y \in Y$ and $\pi \in \mathrm{Aut}(\textsc{Atoms})$. An equivariant function is a function whose graph is an equivariant relation.

Standard set-theoretic relations such as set membership, or set containment, are equivariant. Indeed, $x \in Z \leftrightarrow (\pi \cdot x) \in (\pi \cdot Z)$, etc.

If $\sim$ is an equivariant equivalence relation on $X$ then $\mathrm{Aut}(\textsc{Atoms})$ acts on the set $X/_{\sim}$, by $\pi \cdot C = \{\pi \cdot x \mid x \in C\}$ for each $\sim$-equivalence class $C \subseteq X$.

*Register automata.* Fix a structure $\textsc{Atoms}$ and let $\mathcal{R}$ be an $\mathsf{NRA}$ with input alphabet $S \times \textsc{Atoms}$, control states $Q$, and with $r$ registers. The group $\mathrm{Aut}(\textsc{Atoms})$ acts on all the components of $\mathcal{R}$:

- on the input alphabet $A := S \times \textsc{Atoms}$, via $\pi \cdot (s, a) = (s, \pi(a))$;
- on the set $C := Q \times (\textsc{Atoms} \uplus \{\bot\})^r$ of all configurations of $\mathcal{R}$, via

$$\pi \cdot q(a_1, \ldots, a_r) = q(\pi(a_1), \ldots, \pi(a_r)) \qquad \text{(where } \pi(\bot) = \bot\text{);}$$

- the set of initial configurations and the set of accepting configurations are both equivariant subsets of $C$;
- the set of transitions of $\mathcal{R}$ is an equivariant relation: if $p(\mathbf{a}) \xrightarrow{(s,a)} q(\mathbf{a}')$ is a transition of $\mathcal{R}$, then so is $\pi \cdot p(\mathbf{a}) \xrightarrow{(s,\pi(a))} \pi \cdot q(\mathbf{a}')$.

Furthermore, each of these components is orbit-finite, and each of its elements has a finite support. Using the terminology of [5], this means that register automata are a special case of *orbit-finite automata.*

By equivariance of all the components above, the language $L(\mathcal{R})$ of a register automaton is an equivariant subset of $A^* = (S \times \textsc{Atoms})^*$, considered with the componentwise action of $\mathrm{Aut}(\textsc{Atoms})$ on $A^*$, i.e.

$$\pi \cdot ((s_1, a_1), \ldots, (s_n, a_n)) = ((s_1, \pi \cdot a_1), \ldots, (s_n, \pi \cdot a_n)).$$

*Myhill-Nerode theorem.* In order to prove that a language is deterministically recognizable, we use the following Myhill-Nerode characterization.

For an alphabet $A = S \times \textsc{Atoms}$ and data language $L \subseteq A^*$, consider its Myhill-Nerode equivalence $\sim_L \subseteq A^* \times A^*$, defined by

$$u \sim_L v \qquad \text{if and only if} \qquad uw \in L \leftrightarrow vw \in L \quad \text{for all } w \in A^*.$$

**Theorem 3.** *[5, Thm. 3.8 and Thm. 6.4] Let* $\textsc{Atoms}$ *be oligomorphic and* $L \subseteq (S \times \textsc{Atoms})^*$ *be an equivariant language. Then* $L$ *is deterministically recognizable if and only if* $(S \times \textsc{Atoms})^*/_{\sim_L}$ *is orbit-finite.*

Among other things, this theorem immediately implies that the language from Ex. 2 is not deterministically recognizable, neither for the equality atoms nor for the total order atoms. Indeed, two words are Myhill-Nerode equivalent with respect to that language if and only if they contain the same set of letters. Therefore, the language cannot be deterministically recognizable, since automorphisms of $\textsc{Atoms}$ preserve the number of distinct letters in a word.

## 6 Proof of Theorem 1

In the proof, we will make use of an abstract notion of a split of a family of sets.

For any family $\mathcal{F}$ of subsets of a set $X$, a *split* of $\mathcal{F}$ is a pair $(U, V)$ of sets which partition $X$: $X = U \uplus V$, such that both $U$ and $V$ are *finite* unions of elements of $\mathcal{F}$. Obviously, for any splits to exist, $X = \bigcup \mathcal{F}$ must hold.

In the following lemma, $\textsc{Atoms}$ is the equality atoms.

**Lemma 1.** *For any* $\mathrm{Aut}(\textsc{Atoms})$*-set* $X$ *with finitely supported elements, and any equivariant, orbit-finite family* $\mathcal{F}$ *of finitely supported subsets of* $X$*, the set* $\mathcal{G}$ *of splits of* $\mathcal{F}$ *is orbit-finite. Moreover, a bound on the number of orbits of* $\mathcal{G}$ *and the maximal size of the support of an element in* $\mathcal{G}$ *are computable from the analogous bounds for* $\mathcal{F}$*.*

As should be clear after reading Sec. 5, the set of splits of $\mathcal{F}$ is considered with the natural action of $\mathrm{Aut}(\textsc{Atoms})$: $\pi \cdot (U, V) = (\pi \cdot U, \pi \cdot V)$, where $\pi \cdot W = \{\pi \cdot x \mid x \in W\}$ for $W \subseteq X$.

We will prove Lem. 1 in Sec. 6.2. For now, let us show how the lemma implies Thm. 1.

Let $\mathcal{A}$ and $\mathcal{B}$ be two $\mathsf{NRA}$ over an alphabet $A = S \times \textsc{Atoms}$ such that $L(\mathcal{A})$ and $L(\mathcal{B})$ partition $A^*$. We will show that the Myhill-Nerode equivalence of $L = L(\mathcal{A})$ has orbit-finitely many classes. Together with Thm. 3, this will prove that $L$ is deterministically recognizable.

Let $C$ be the set of configurations of $\mathcal{A} \uplus \mathcal{B}$ (the disjoint union of $\mathcal{A}$ and $\mathcal{B}$.) Hence, $C$ consists of tuples of the form $q(\mathbf{a})$ where $q$ is either a state of $\mathcal{A}$ or a state of $\mathcal{B}$ (but not both), and $\mathbf{a}$ is a tuple of elements of $\textsc{Atoms} \uplus \{\perp\}$ of appropriate length. For $c \in C$ denote

$$L_c := \{w \in A^* \mid \mathcal{A} \uplus \mathcal{B} \text{ accepts } w \text{ from configuration } c\},$$

and let $\mathcal{F} = \{L_c \mid c \in C\}$. Since $C$ is equivariant and orbit-finite, so is $\mathcal{F}$. Moreover, if $c = q(\mathbf{a})$ then $L_c$ is finitely supported by the atoms in $\mathbf{a}$. Clearly, every word $(s_1, a_1) \cdots (s_n, a_n) \in A^*$ is supported by $\{a_1, \ldots, a_n\}$. This means that $\mathcal{F}$ and $X = A^*$ satisfy the assumptions of Lem. 1, therefore $\mathcal{F}$ has only orbit-finitely many splits.

Every word $v \in A^*$ induces a partition of $A^*$ into two disjoint sets:

$$U_v = \{w \in A^* \mid vw \in L\} \qquad \text{and} \qquad V_v = \{w \in A^* \mid vw \notin L\}\,.$$

Moreover, the sets $U_v$ and $V_v$ are finite unions of sets from $\mathcal{F}$, namely

$$U_v = \bigcup_{c \in \mathcal{A}(v)} L_c \qquad \text{and} \qquad V_v = \bigcup_{c \in \mathcal{B}(v)} L_c.$$

These unions are finite because automata $\mathcal{A}$ and $\mathcal{B}$ allow no guessing and so $\mathcal{A}(v)$ and $\mathcal{B}(v)$, the sets of configurations reachable in $\mathcal{A}$ resp. $\mathcal{B}$ by reading the word $v$, are finite. Therefore, $(U_v, V_v)$ is a split of $\mathcal{F}$, for any word $v$.

By definition, $u \sim_L v$ if and only if $U_u = U_v$. Consider any two words $v, w \in A^*$ such that the splits $(U_v, V_v)$ and $(U_w, V_w)$ are in the same orbit, i.e., $U_w = \pi \cdot U_v$ (and therefore also $V_w = \pi \cdot V_v$) for some automorphism $\pi$. Since $L$ is an equivariant language, we have $\pi \cdot U_v = U_{\pi \cdot v}$ and so $w \sim_L \pi \cdot v$. Theorem 1 now follows from Thm. 3.

### 6.1 Examples

Before proving Lem. 1, we give some examples of families of splits, which may be helpful in developing some intuitions.

The first example shows that the number of orbits of splits may grow as fast as double-exponentially, relative to the least supports of elements of $\mathcal{F}$.

*Example 6.* For the equality atoms, fix $k \geqslant 1$ and let $X$ be the set of all $k$-tuples of pairwise distinct atoms. For each $S \subseteq \textsc{Atoms}$ with $|S| = k$, let $S^{(k)} = S^k \cap X$ and let $M_S = X \setminus S^{(k)}$. Note that $S^{(k)}$ is finite, with $k!$ elements.

The family $\mathcal{F} \subseteq \mathcal{P}(X)$ of all singletons in $X$ and all sets $M_S$ as above is equivariant and has two orbits. Each set in $\mathcal{F}$ has a support of size $k$.

For any $K \subseteq S^{(k)}$, consider the partition of $X$ into $K$ and $X \setminus K$. Then $(K, X \setminus K)$ is a split of $\mathcal{F}$, as $K = \bigcup_{v \in K} \{v\}$ and $X \setminus K = M_S \cup \bigcup_{v \in S^{(k)} \setminus K} \{v\}$.

Moreover, every split $(U, V)$ of $\mathcal{F}$ is of the form $(K, X \setminus K)$ or $(X \setminus K, K)$ for some $S$ and $K$ as above. Indeed, suppose $U = \bigcup \mathcal{U}$ and $V = \bigcup \mathcal{V}$ for some finite $\mathcal{U}, \mathcal{V} \subseteq \mathcal{F}$. As $U \cup V = X$ is infinite, $\mathcal{U} \cup \mathcal{V}$ must contain $M_S$ for some set $S$ of $k$ atoms. Suppose without loss of generality that $M_S \in \mathcal{U}$. By disjointness of $U$ and $V$, the set $\mathcal{V} \subseteq \mathcal{F}$ may only contain singletons $\{v\}$, for $v \in S^{(k)}$. Then $(U, V) = (X \setminus K, K)$, where $K = \bigcup \mathcal{V}$.

For $K, K' \subseteq S^{(k)}$, the splits defined by $K$ and $K'$ are in the same orbit only if there is an automorphism $\pi$ that fixes $S$ as a set, such that $\pi \cdot K = K'$. Since there are only $k!$ bijections on $S$, the set of splits of $\mathcal{F}$ has at least $\frac{2^{k!}}{k!}$ orbits. $\square$

The next example shows the difference between splits and the finite subfamilies of $\mathcal{F}$ that define those splits: the set of those families may be orbit-infinite.

*Example 7.* Let $X$ be the set of all finite sets of equality atoms. For any distinct atoms $a, b$, define $E_{a,b}, D_{a,b} \subseteq X$ by:

$$E_{a,b} = \{F \in X \mid a \in F \leftrightarrow b \in F\} \qquad D_{a,b} = X \setminus E_{a,b}$$

And let $\mathcal{F}$ contain all sets $E_{a,b}$ and $D_{a,b}$. This $\mathcal{F}$ has two orbits.

Obviously, $(U, V) = (X, \emptyset)$ is a split of $\mathcal{F}$; it is enough to take $\mathcal{U} = \{D_{a,b}, E_{a,b}\}$ and $\mathcal{V} = \emptyset$ for any fixed $a, b$. However, there are many more minimal families $\mathcal{U}$ and $\mathcal{V}$ that achieve the same effect. Indeed, for any number $n$, and for any pairwise distinct atoms $a_1, \ldots, a_n$, consider:

$$\mathcal{U} = \{D_{a_1,a_2}, D_{a_2,a_3}, \ldots, D_{a_{n-1},a_n}, E_{a_1,a_n}\} \qquad \mathcal{V} = \emptyset$$

It is easy to check that $\bigcup \mathcal{U} = X$. All such families are minimal (in fact, removing any element from $\mathcal{U}$ would prevent it from being the part of any split of $\mathcal{F}$), and for each $n$ these families form a separate orbit. □

The following example shows that the statement of Lem. 1 fails if the atoms are $(\mathbb{Q}, \leqslant)$. It is obtained from Ex. 4 via the translation given in the proof of Thm. 1, and a simplification replacing each word by its last letter.

*Example 8.* The atoms are $(\mathbb{Q}, \leqslant)$. Let $X = \mathbb{Q}$ and let $\mathcal{F} \subseteq \mathcal{P}(X)$ consist of:

- singletons $\{q\} \subseteq X$, for $q \in \mathbb{Q}$;
- open intervals $(p, q) \subseteq X$, for $p < q$ in $\mathbb{Q} \cup \{-\infty, +\infty\}$.

Then $\mathcal{F}$ has five orbits (here $\pm\infty$ are fixed under the action of Aut(Atoms)). For any finite set $K \subseteq X$, consider the partition of $X$ into $K$ and $X \setminus K$. Then $K = \bigcup_{q \in K} \{q\}$ whereas $X \setminus K$ is the union of all intervals $(p, q)$, where $p < q$ are consecutive elements in $K \cup \{-\infty, +\infty\}$. Hence, $(K, X \setminus K)$ is a split of $\mathcal{F}$. In particular, the set of all splits of $\mathcal{F}$ has infinitely many orbits, because the set of finite subsets of $X$ has infinitely many orbits. □

### 6.2 Proof of Lemma 1

We prove by induction a stronger statement, where the atoms are assumed to be an expansion of $(\mathbb{N}, =)$ by finitely many constants. In other words, in this section we will assume that Atoms is a structure over a vocabulary that consists of (equality and) a finite number of constant symbols; the universe of Atoms is $\mathbb{N}$, with the constants interpreted as some pairwise distinct numbers. The group Aut(Atoms) then consists of all bijections of Atoms which fix every constant.

If Atoms is such a structure and $T$ is a finite set of atoms all different from the constants, then by $\text{Atoms}_T$ we denote the structure, over an extended vocabulary, that arises from Atoms by interpreting all the atoms in $T$ as additional constants. Obviously, $\text{Aut}(\text{Atoms}_T)$ is a subgroup of Aut(Atoms), so every action of Aut(Atoms) on a set $X$ restricts to an action of $\text{Aut}(\text{Atoms}_T)$.

This restriction preserves and reflects the existence of finite supports: an element $x \in X$ is supported by some $S$ in the action of $\mathrm{Aut}(\textsc{Atoms})$ if and only if it is supported by $S \setminus T$ in the restricted action of $\mathrm{Aut}(\textsc{Atoms}_T)$. In particular, if Atoms is an expansion of $(\mathbb{N}, =)$ by finitely many constants, then every finitely supported element $x$ has a least support $\sup(x)$. Note that $\sup(x)$ never contains any constants, since those can always be safely removed from any support.

For a subset $\mathcal{U}$ of an orbit-finite equivariant set $\mathcal{F}$, its *dimension* $\dim(\mathcal{U})$ is the maximum size of the least support of an element of $\mathcal{U}$. This makes sense even if $\mathcal{U}$ is infinite, because $\mathcal{F}$ is orbit-finite and sets from the same orbit have least supports of the same size. In particular, $\dim(\mathcal{F})$ is well defined.

The following lemma says that adding constants to atoms preserves orbit-finiteness. It is a standard result in the theory of sets with atoms, see e.g. [1, Lem. 3.19] or [24, Lem. 5.22], indeed it is a fundamental property of oligomorphic structures, but we re-prove it here to extract explicit bounds:

**Lemma 2.** *Fix a finite set $T \subseteq \textsc{Atoms}$. For any orbit-finite $\mathrm{Aut}(\textsc{Atoms})$-set $\mathcal{F}$ with $l$ orbits, the corresponding action of $\mathrm{Aut}(\textsc{Atoms}_T)$ on $\mathcal{F}$ is also orbit-finite, with at most $l \cdot (|T|+1)^{\dim(\mathcal{F})}$ orbits.*

*Proof.* Assume first that $\mathcal{F}$ has only one orbit in the $\mathrm{Aut}(\textsc{Atoms})$-action, i.e., that $l = 1$. Let $d = \dim(\mathcal{F})$. Let $Y$ denote the set of $d$-tuples of pairwise distinct atoms different from the constants in Atoms. This is a single-orbit set under the componentwise action of $\mathrm{Aut}(\textsc{Atoms})$. Pick any $x_0 \in \mathcal{F}$. Let $y_0 = (a_1, \ldots, a_d) \in Y$ be an enumeration of $\sup(x_0)$. There is a unique equivariant surjection $f \colon Y \to X$ such that $f(\pi \cdot y_0) = \pi \cdot x_0$ for all $\pi \in \mathrm{Aut}(\textsc{Atoms})$. (The function $f$ is total since $Y$ has one orbit; it is well defined because $y_0$ enumerates a support of $x_0$, and it is surjective since $X$ has one orbit.) Two tuples in $Y$ are in the same orbit in the action of $\mathrm{Aut}(\textsc{Atoms}_T)$ if and only if they contain the same arrangement of atoms from $T$ at the same positions. There are at most $(|T|+1)^d$ such arrangements, (in fact fewer than this if $d > 1$, because tuples in $Y$ are pairwise distinct), so $Y$ has at most $(|T|+1)^d$ such orbits. $X$ is an image of the equivariant function $f \colon Y \to X$, so the same bound applies to $X$. For a set $\mathcal{F}$ with $l$ orbits, each of dimension at most $d$, the bound simply multiplies by $l$. $\square$

From now on consider Atoms as described above, and let $X$ and $\mathcal{F}$ be as in the statement of Lem. 1. The following key lemma says that every split of $\mathcal{F}$ has a support of a bounded size.

**Lemma 3.** *Let $U \uplus V$ be a split of $\mathcal{F}$ and let $\mathcal{U}, \mathcal{V}$ be finite subfamilies of $\mathcal{F}$ such that $\bigcup \mathcal{U} = U$ and $\bigcup \mathcal{V} = V$. Then $U$ and $V$ each have a support of size at most $N$, for some bound $N$ computable only from $\dim(\mathcal{U}), \dim(\mathcal{V}), \dim(\mathcal{F})$ and the number of orbits in $\mathcal{F}$.*

The crux of this lemma is that the number $N$ does not depend on the split $U \uplus V$. It only depends on the number of orbits in $\mathcal{F}$, its dimension $\dim(\mathcal{F})$, and on $\dim(\mathcal{U})$ and $\dim(\mathcal{V})$ (which, anyway, are bounded from above by $\dim(\mathcal{F})$).

*Proof (of Lem. 3).* We proceed by induction on $k = \dim(\mathcal{U}) + \dim(\mathcal{V})$. Fix $k \geqslant 0$ and assume that the statement of the lemma holds for all smaller values of $k$. Without loss of generality, we may assume that $\emptyset$ does not belong to $\mathcal{U}$ nor $\mathcal{V}$ (as it can be safely removed from each of them).

For a finitely supported set $F \subseteq X$ define

$$F^\sharp := \{\pi \cdot y \mid \pi \in \mathrm{Aut}(\textsc{Atoms}), y \in F, \sup(y) \cap \sup(F) = \emptyset\}.$$

Intuitively, $F^\sharp$ arises by taking all elements of $F$ that are "fresh for $F$", i.e., ones whose supports share no atoms with the support of $F$, and then by applying arbitrary atom automorphisms to those elements. Note that that $F^\sharp$ is equivariant and $F^\sharp = (\pi \cdot F)^\sharp$ for any automorphism $\pi$.

**Claim 1** $X = \bigcup_{F \in \mathcal{U} \cup \mathcal{V}} F^\sharp$.

*Proof.* Take any $x \in X$. Let $S = \bigcup_{F \in \mathcal{U} \cup \mathcal{V}} \sup(F)$. Since $\mathcal{U}$ and $\mathcal{V}$ are finite, $S$ is a finite set. Pick an automorphism $\pi$ such that its inverse $\pi^{-1}$ maps $\sup(x)$ to a set disjoint with $S$. Consider the element $y = \pi^{-1} \cdot x \in X$. Since $U \cup V = X$, there must be some $F \in \mathcal{U} \cup \mathcal{V}$ such that $y \in F$. Then $x \in F^\sharp$. □

Let us first prove the lemma for the **special case** where $X = F^\sharp$ for some $F \in \mathcal{U} \cup \mathcal{V}$. Suppose that $X = F^\sharp$ for some $F \in \mathcal{U}$ (the case $F \in \mathcal{V}$ is symmetric).

**Claim 2** Every $y \in X$ with $\sup(y) \cap \sup(F) = \emptyset$ belongs to $F$.

*Proof.* Take any $y$ as above. As $X = F^\sharp$, there is some $\pi$ and $x \in F$ such that $y = \pi \cdot x$ and $\sup(x) \cap \sup(F) = \emptyset$. Pick an automorphism $\theta$ such that:

– $\theta$ agrees with $\pi$ on $\sup(x)$, mapping it bijectively to $\sup(y)$,
– $\theta$ fixes $\sup(F)$ pointwise.

Such a $\theta$ exists since $\sup(x)$ and $\sup(y)$ are both disjoint from $F$. Then $\theta \cdot x = \pi \cdot x = y$ by the first property above, and $\theta \cdot x \in \theta \cdot F = F$ by the second property. Altogether, $y \in F$. □

**Claim 3** For every $G \in \mathcal{V}$, $\sup(F) \cap \sup(G) \neq \emptyset$.

*Proof.* We show that if $\sup(G)$ is disjoint from $\sup(F)$ then $G$ must be empty, contradicting our previous assumption.

Suppose $x \in G$. Pick an automorphism $\pi$ which fixes $\sup(G)$ pointwise and maps $\sup(x)$ to a set disjoint with $\sup(F)$. Such a $\pi$ exists because $\sup(G)$ and $\sup(F)$ are disjoint. Letting $y := \pi \cdot x$, we have $y \in F$ by Claim 2, and moreover $y = \pi \cdot x \in \pi \cdot G = G$. Then $y \in F \cap G \subseteq U \cap V = \emptyset$, a contradiction. This proves $G = \emptyset$, which in turn contradicts the assumption that $\emptyset \notin \mathcal{V}$. □

Denote $T = \sup(F)$. If $T = \emptyset$ then by Claim 3, $\mathcal{V}$ has dimension 0 and therefore $V$ is supported by the empty set. So we may assume that $T \neq \emptyset$. For the same reason we may assume that the family $\mathcal{V}$ is not empty.

Let $\text{Atoms}_T$ be obtained from Atoms by including the elements of $T$ as new constants. Hence, $\text{Atoms}_T$ extends Atoms by at most $r$ constants, where $r := \dim(\mathcal{F})$.

Let $l$ be the number of orbits in $\mathcal{F}$. By Lem. 2, the family $\mathcal{F}$, treated as a family of sets over the atoms $\text{Atoms}_T$, is still orbit-finite, with the number of orbits $l'$ depending only on $l$ and $r$. Clearly, $U \uplus V$ remains a split of $\mathcal{F}$. Note that if $F \in \mathcal{F}$ is supported by some set $S$ over Atoms, then $F$ is supported by $S$, indeed even by $S \setminus T$, over $\text{Atoms}_T$. In particular, the dimension of $\mathcal{F}$ does not increase by moving from Atoms to $\text{Atoms}_T$. More interestingly, by Claim 3, the least supports of all the elements in $\mathcal{V}$ actually *decrease* when considering $\text{Atoms}_T$ as atoms. Since $\mathcal{V}$ is not empty, the dimension of $\mathcal{V}$ strictly decreases and it follows that $\dim(\mathcal{U}) + \dim(\mathcal{V}) < k$ over $\text{Atoms}_T$. Applying the inductive assumption yields a set $T'$ of size $N'$, depending on $k-1$ and $l'$, such that $T'$ supports $V$ over $\text{Atoms}_T$. By construction, $V$ is supported by $T \cup T'$ over Atoms. Note that

$$|T \cup T'| \leqslant N'' := N' + r.$$

This concludes the proof in the special case when $X = F^\sharp$ for some $F \in \mathcal{U} \cup \mathcal{V}$. In the **general case**, for each $F \in \mathcal{U} \cup \mathcal{V}$ define:

$$\mathcal{F}_F := \{G \cap F^\sharp \mid G \in \mathcal{F}\}$$

$$\mathcal{U}_F := \{G \cap F^\sharp \mid G \in \mathcal{U}\} \qquad \mathcal{V}_F := \{G \cap F^\sharp \mid G \in \mathcal{V}\}$$
$$U_F := U \cap F^\sharp = \bigcup \mathcal{U}_F \qquad V_F := V \cap F^\sharp = \bigcup \mathcal{V}_F.$$

Then $\bigcup \mathcal{F}_F = F^\sharp$ and $(U_F, V_F)$ is a split of $\mathcal{F}_F$ which falls into the special case considered above. Hence, $U_F$ has some support $S_F$ of size at most $N''$.

Then $U$ is supported by $S := \bigcup_{F \in \mathcal{U} \cup \mathcal{V}} S_F$. Note that $S_F$ only depends on the orbit of $F$, as $F^\sharp = (\pi \cdot F)^\sharp$ for any automorphism $\pi$. As there are $l$ such orbits contained in $\mathcal{F}$, it follows that $S$ has size at most $N := N''l$. This concludes the inductive step, and the proof of Lem. 3. □

Using Lem. 3, we now proceed to prove Lem. 1.

*Proof (of Lemma 1).* Consider an equivariant set $X$ and an equivariant, orbit finite family $\mathcal{F}$ of finitely supported subsets of $X$. Let $((U_i, V_i))_{i \in I}$ be a family of splits of $\mathcal{F}$. By Lem. 3, each one of these splits is supported by some set of a bounded size. Applying suitable automorphisms to each of these splits, we can obtain a family of splits $((U'_i, V'_i))_{i \in I}$ such that, for all $i \in I$:

- $U'_i$ and $U_i$ are in the same orbit, and
- each $U'_i$ is supported by the same set $S$.

It is now enough to show that there are only finitely many subsets $U \subseteq X$ supported by a fixed set $S$, which are unions of elements of $\mathcal{F}$.

By Lem. 2 it follows that $\mathcal{F}$ has finitely many orbits under the action of the group $\text{Aut}(\text{Atoms}_S)$ of all automorphisms which fix $S$ pointwise. (Here, as in the statement of Lem. 1, Atoms are the pure equality atoms without any constants.) If a set $U \subseteq X$ supported by $S$ contains some $F \in \mathcal{F}$ as a subset, then it contains $\pi \cdot F$ for every $\pi \in \mathrm{Aut}(\mathrm{Atoms}_S)$. In other words, $U$ contains (the union of) the entire orbit in $\mathcal{F}$ under the action of $\mathrm{Aut}(\mathrm{Atoms}_S)$. Since we assume that $U$ is a union of elements of $\mathcal{F}$, it is a union of (the unions of) orbits in $\mathcal{F}$, and there are only finitely many of these.

This completes the proof of Lem. 1. □

## 7 Application to Unambiguous Register Automata

Lemma 1 is interesting in its own right and its applications are not limited to the ones mentioned in Sec. 4. We shall now show how it can be used to decide universality (and hence also language containment and equality, cf. [8, Lem. 8]) of URA over the pure equality atoms Atoms.

**Theorem 4.** *[22, Thm. 14] The language containment and equality problems are decidable for unambiguous register automata.*

As an application of Lem. 1, we give an alternative decidability proof for the universality problem of URA. First, we prove a consequence of Lem. 1.

**Lemma 4.** *Let $X$ be an equivariant set over equality atoms, and let $\mathcal{F}$ be an equivariant, orbit-finite family of finitely supported subsets of $X$. There is a bound $M$, computable from* $\dim(\mathcal{F})$ *and the number of orbits in $\mathcal{F}$, such that every $\mathcal{P} \subseteq \mathcal{F}$ which is a partition of $X$ has size at most $M$.*

*Proof.* Let $\mathcal{G} = \{U \mid (U, V) \text{ is a split of } \mathcal{F}\}$. By Lem. 1, $\mathcal{G}$ is orbit-finite. Moreover, its elements are finitely supported. Let $\mathcal{P} \subseteq \mathcal{F}$ be a partition of $X$ into nonempty subsets. For each $\mathcal{U} \subseteq \mathcal{P}$, the union $\bigcup \mathcal{U}$ belongs to $\mathcal{G}$; in particular, we have $2^{|\mathcal{P}|}$ elements of $\mathcal{G}$, each containing different sets in $\mathcal{P}$. The proof is completed by the following counting argument.[1]

Let $S = \bigcup_{F \in \mathcal{P}} \sup(F)$. An *$S$-orbit* in $\mathcal{G}$ is an orbit in $\mathcal{G}$ with respect to the action of those atom permutations which fix $S$ pointwise. Equivalently, it is an orbit in $\mathcal{G}$ viewed as a $\mathrm{Aut}(\mathrm{Atoms}_S)$-set. By Lem. 2, for any finite $S \subseteq \mathrm{Atoms}$, the number of $S$-orbits in $\mathcal{G}$ is bounded by $l \cdot (|S| + 1)^k$, where $k$ and $l$ are computable from $\dim(\mathcal{F})$ and the number of orbits of $\mathcal{F}$.

Two splits $G, G' \in \mathcal{G}$ in the same $S$-orbit contain the same elements of $\mathcal{P}$: if $G' = \pi \cdot G$ then by equivariance of $\mathcal{F}$ and $\mathcal{G}$, for each $F \in \mathcal{P}$ we have $F \subseteq G$ if and only if $\pi \cdot F \subseteq \pi \cdot G$, but $\pi \cdot F = F$ when $\pi$ fixes $S$ pointwise. Hence, for any two distinct $\mathcal{U}, \mathcal{U}' \subseteq \mathcal{P}$, their unions $\bigcup \mathcal{U}$ and $\bigcup \mathcal{U}'$ belong to different $S$-orbits in $\mathcal{G}$, so there are least $2^{|\mathcal{P}|}$ such orbits. As $|S| \leqslant \dim(\mathcal{F}) \cdot |\mathcal{P}|$, we get:

$$2^{|\mathcal{P}|} \leqslant l \cdot (|S| + 1)^k \leqslant l \cdot (\dim(\mathcal{F}) \cdot |\mathcal{P}| + 1)^k.$$

It follows that $|\mathcal{P}|$ is bounded by some $M$ computable from $k, l$, and $\dim(\mathcal{F})$. □

[1] It exhibits the well-known fact that equality atoms have the *NIP property* studied in model theory.

Lemma 4 has the following corollary, which is a strong restriction on the structure of universal URA and easily yields Thm. 4.

Call a configuration $c$ of a NRA $\mathcal{A}$ *nonempty* if the NRA accepts some word from this configuration, i.e., the following language is nonempty:

$$L_c := \{w \in A^* \mid \mathcal{A} \text{ accepts } w \text{ from } c\}$$

Since NRA emptiness is decidable, it is not difficult to modify any given NRA to one with only nonempty configurations. This transformation preserves URA, so we may safely assume that we only consider URA with this property.

**Corollary 1.** *Let $\mathcal{A}$ be a* URA *with nonempty configurations and which accepts every input word. Then there is a computable bound $M$ such that $\mathcal{A}$ may reach at most $M$ different configurations when reading any given input word.*

*Proof.* Let $\mathcal{A}$ be an URA over an input alphabet $A = S \times \text{Atoms}$. Let $C$ be the set of configurations of $\mathcal{A}$ and let $\mathcal{F} := \{L_c \mid c \in C\}$. Note that $\dim(\mathcal{F})$ is not larger than the number of registers $r$ of $\mathcal{A}$, and the number of orbits in $\mathcal{F}$ is not larger than the number of orbits of configurations in $\mathcal{A}$, which in turn is equal to the number of control states in $\mathcal{A}$ times the number of orbits in $(\text{Atoms} \uplus \{\bot\})^r$ (equal to the $r+1$-st Bell number).

For each $w \in A^*$, the set $\mathcal{A}(w) \subseteq C$ of configurations reachable when reading $w$ is finite, since $\mathcal{A}$ has no guessing. Unambiguity of $\mathcal{A}$ implies that the family

$$\mathcal{P}_w := \{L_c \mid c \in \mathcal{A}(w)\} \subseteq \mathcal{F}$$

consists of pairwise disjoint sets. If additionally $L(\mathcal{A}) = A^*$, then $\mathcal{P}_w$ forms a partition of $A^*$, so $|\mathcal{P}_w| \leqslant M$ where $M$ is the bound from Lemma 4. As $|\mathcal{A}(w)| \leqslant |\mathcal{P}_w|$, this yields the corollary. □

Decidability of universality of URA now follows using standard ideas.

*Proof (of Thm. 4, sketch).* We use the notation of the proof of Cor. 1. The idea is to construct the truncated powerset automaton whose states are sets of at most $M$ states of $\mathcal{A}$.

Let $C'$ denote the family of subsets of $C$ of size at most $M$; then $C'$ is orbit-finite. We define a deterministic automaton $\mathcal{A}'$ with an infinite, but orbit-finite state space $C'$. Its transitions are $X \xrightarrow{a} Y$, for $X, Y \in C'$ such that

$$Y = \left\{ y \in C \;\middle|\; x \xrightarrow{a} y \text{ in } \mathcal{A}, x \in X \right\}.$$

The initial state of $\mathcal{A}'$ is the set $C_0 \subseteq C$ of initial configurations of $\mathcal{A}$ (unless $|C_0| > M$, but then $L(\mathcal{A}) \neq A^*$ by the corollary). Accepting states are all states $X \in C'$ which contain an accepting configuration of $\mathcal{A}$. All the ingredients of $\mathcal{A}'$ are equivariant, orbit-finite sets, so $\mathcal{A}'$ is an *orbit-finite deterministic automaton*, and can be effectively constructed given $\mathcal{A}$ and $M$. Its language $L(\mathcal{A}')$ is defined as usual. By construction,

- $L(\mathcal{A}') \subseteq L(\mathcal{A}) \subseteq A^*$;

– if $L(\mathcal{A}) = A^*$ then $L(\mathcal{A}') = A^*$, by Cor. 1.

Hence, $\mathcal{A}'$ is universal if and only if $\mathcal{A}$ is universal. Since $\mathcal{A}'$ is orbit-finite, universality of $\mathcal{A}'$ can be effectively decided, using standard techniques for orbit-finite automata [1,5]: by first complementing and then testing emptiness. □

## 8 Proof of Theorem 2

Towards proving Thm. 2, assume $\mathcal{A}$ and $\mathcal{B}$ are two complementing 1-NRA over an alphabet $A = S \times \text{Atoms}$ and that Atoms admit wqo.

Recall that configurations of a 1-NRA are either of the form $q(a)$ where $q$ is a control state and $a \in \text{Atoms}$ is the register value, or of the form $q(\perp)$ when the register value is still undefined. We assume, without losing generality, that both register automata $\mathcal{A}$ and $\mathcal{B}$ immediately update their register, i.e., every transition rule outgoing from an initial state updates the register.

Let $Q$ and $Q'$ denote sets of control states of $\mathcal{A}$ and $\mathcal{B}$, respectively, and assume without losing generality that $Q$ and $Q'$ are disjoint.

For every nonempty data word $w \in A^+$, the set $\mathcal{A}(w) \cup \mathcal{B}(w)$ of configurations of $\mathcal{A}$ and $\mathcal{B}$ reachable along $w$ is finite, since NRA have no guessing, and contains no undefined configurations of the form $q(\perp)$ due to the immediate update assumption. For every $w \in A^+$ define a finite induced substructure $\mathcal{C}_w$ of Atoms, labeled with the finite set $P = \mathcal{P}(Q \cup Q')$, as follows. The elements of $\mathcal{C}_w$ are the atoms that appear in configurations in $\mathcal{A}(w) \cup \mathcal{B}(w)$:

$$\mathcal{C}_w = \{a \in \text{Atoms} \mid (q, a) \in \mathcal{A}(w) \cup \mathcal{B}(w) \text{ for some state } q.\}$$

The labeling $\ell_w \colon \mathcal{C}_w \to P$ of $\mathcal{C}_w$ maps $a \in \mathcal{C}_w$ to the set of all control states which appear in $\mathcal{A}(w) \cup \mathcal{B}(w)$ together with $a$:

$$\ell_w(a) = \{q \in Q \mid (q, a) \in \mathcal{A}(w)\} \cup \{q \in Q' \mid (q, a) \in \mathcal{B}(w)\}\,.$$

Let $L = L(\mathcal{A})$. For each $v \in A^*$ define the partition of $A^*$ into:

$$U_v = \{w \in A^* \mid vw \in L\} \qquad \text{and} \qquad V_v = \{w \in A^* \mid vw \notin L\}\,.$$

Recall that $u \sim_L v$ if and only if $U_u = U_v$.

*Claim.* Let $u, v \in A^+$. If $\mathcal{C}_u \preceq \mathcal{C}_v$ then $\pi \cdot u \sim_L v$ for some automorphism $\pi$.

*Proof.* By definition of $\preceq$, there is some $\pi \in \text{Aut}(\text{Atoms})$ which maps $\mathcal{C}_u$ to a substructure of $\mathcal{C}_v$, so that $\pi \cdot \mathcal{C}_u \subseteq \mathcal{C}_v$ and

$$\ell_u(a) = \ell_v(\pi(a)) \qquad \text{for } a \in \mathcal{C}_u. \tag{2}$$

Let $u' = \pi \cdot u$. By equivariance of register automata, if $\mathcal{A}$ reaches a configuration $(q, a)$ when reading $u$, then it reaches the configuration $(q, \pi(a))$ when reading $u' = \pi \cdot u$. Hence, $\mathcal{C}_{u'} \subseteq \mathcal{C}_v$ and $\ell_u(a) = \ell_{u'}(\pi(a))$ for $a \in \mathcal{C}_u$. Together with (2) we get $\ell_{u'}(a) = \ell_v(a)$ for all $a \in \mathcal{C}_{u'}$.

We show that this implies $U_{u'} = U_v$, which will yield the claim as $u' = \pi \cdot u$. Towards proving $U_{u'} \subseteq U_v$ take any $w \in U_{u'}$; then $u'w \in L$. Pick an accepting run of $\mathcal{A}$ on $u'w$. Let $q(a)$ be the configuration of $\mathcal{A}$ in this run reached after reading the (nonempty) prefix $u'$. In particular, $\mathcal{A}$ accepts $w$ starting from the configuration $q(a)$. Moreover, $a \in \mathcal{C}_{u'}$ and $q \in \ell_u(a)$. As $\mathcal{C}_{u'} \subseteq \mathcal{C}_v$ and $\ell_{u'}(a) = \ell_v(a)$, it follows that $\mathcal{A}$ may reach the configuration $q(a)$ after reading $v$. As $w$ is accepted by $\mathcal{A}$ from this configuration, it follows that $\mathcal{A}$ accepts $vw$, so $w \in U_v$.

The inclusion $V_{u'} \subseteq V_v$ is proved by a similar argument, using $\mathcal{B}$ instead of $\mathcal{A}$, since $L(\mathcal{B}) = A^* \setminus L(\mathcal{A}) = A^* \setminus L$. As $U_{u'} = A^* \setminus V_{u'}$ and $V_v = A^* \setminus U_v$, the inclusion $V_{u'} \subseteq V_v$ implies $U_{u'} \supseteq U_v$. Altogether, $U_{u'} = U_v$, so $u' \sim_L v$, yielding the claim. □

Theorem 2 now follows easily: assume towards a contradiction that $A^*/_{\sim_L}$ is not orbit-finite. Then there is an infinite set $X \subseteq A^+$ such that $\pi(u) \not\sim_L v$ for all distinct $u, v \in X$ and $\pi \in \mathrm{Aut}(\textsc{Atoms})$. As Atoms admits wqo, there are distinct $u, v \in X$ such that $\mathcal{C}_u \preceq \mathcal{C}_v$. The claim above yields a contradiction. □

## 9 Final remarks

We have studied a deterministic collapse for NRA: if a language and its complement are both recognized by NRA then they are also recognized by DRA. We have proved this for register automata over equality atoms; and for automata with one register only, over any atoms that admit wqo. We have also applied our key technical observation, namely orbit-finiteness of the set of splits of an orbit-finite family of sets, in order to re-prove decidability of universality of URA.

The assumed form $A = S \times \textsc{Atoms}$ of the input alphabets is not important; the results apply to arbitrary orbit-finite input alphabets $A$.

The proof of our main result (also of decidability of universality of URA) is effective, with elementary bounds. In particular, given two NRA with complementing languages the equivalent DRA from Thm. 1 has an exponential number of registers and a doubly-exponential number of orbits of states. The same bounds apply to a DRA constructed in our proof of Thm. 4. Moreover, assuming Atoms satisfy standard effectiveness assumptions, like decidability of their first-order theory, one can also compute an equivalent DRA from Thm. 2.

Concerning possible generalisations of our results, we believe that Thm. 1 holds not only for equality atoms, but for arbitrary oligomorphic $\omega$-stable atoms. These include e.g. the nested equality atoms mentioned in Sec. 2. On the other hand Thm. 1 does not extend to disjoint but non-complementing NRA languages: it is not true that for every two disjoint NRA languages there is a DRA language that *separates* them, i.e., includes one of them and is disjoint from the other. The corresponding decision problem (given two disjoint NRA, does a separating DRA exist?) is decidable when the number of registers of a separating automaton is fixed [9], and open in general.

An intriguing open question (not unlike the wqo Dichotomy Conjecture [19]) is whether it is necessary for Atoms to admit wqo for Thm. 2 to hold.
